# A practical approach to a single-chain variable fragment (scFv) biotinylating for immunohistochemical analysis^✰^

**DOI:** 10.1016/j.mex.2026.103989

**Published:** 2026-06-05

**Authors:** Cynthia Rodríguez-Nava, Carlos Ortuño-Pineda, Amalia Vences-Velázquez, Berenice Illades-Aguiar, Karen Cortés-Sarabia, Luz del Carmen Alarcón-Romero

**Affiliations:** a Laboratorio de Investigación en Citopatología e Histoquímica, Universidad Autónoma de Guerrero, Chilpancingo Guerrero, Mexico; b Laboratorio de Ácidos Nucleicos y Proteínas, Universidad Autónoma de Guerrero, Chilpancingo Guerrero, Mexico; c Laboratorio de Investigación en Inmunobiología y Diagnóstico Molecular, Universidad Autónoma de Guerrero, Chilpancingo Guerrero, Mexico; d Laboratorio de Investigación en Biomedicina Molecular, Universidad Autónoma de Guerrero, Chilpancingo Guerrero, Mexico

**Keywords:** Single-chain variable fragment (scFv), Biotinylation, Immunohistochemistry

## Abstract

Immunohistochemistry (IHC) is a widely used technique for detecting proteins in tissue sections; however, its performance is significantly influenced by the quality of antibodies and detection strategies. Conventional IgG or IgM-based antibodies often come with high production costs, variability between batches, and limited penetration into tissues, which can compromise the uniformity and reproducibility of staining. Recombinant single-chain variable fragments (scFvs) offer an attractive alternative due to their smaller size, defined molecular composition, and renewable production capabilities. In this work, we focus on optimizing IHC by chemically biotinylating scFvs, allowing for direct coupling to streptavidin-based detection systems. This method enhances signal sensitivity while eliminating the need for secondary antibodies or additional labeling steps. Biotinylated scFvs can diffuse more easily into tissue sections, streamline the staining workflow, and reduce both assay time and cost. Overall, integrating biotinylated scFvs into IHC protocols presents a robust, scalable, and reproducible strategy that addresses key limitations of conventional antibody-based methods, thus supporting more consistent and efficient protein detection in histological analyses.This protocol allowed the optimization of conventional IHC conditions by using biotinylating scFv to achieve precise, time and cost-efficient antigen visualization.

## Graphical abstract

**Figure:**
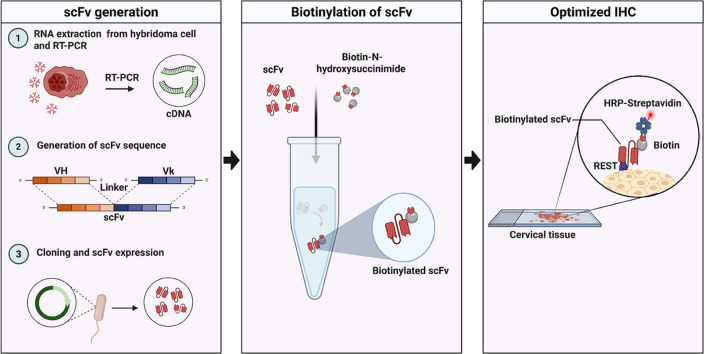

**Specifications table**

**Table:** 

**Subject area**	Immunology and Microbiology
**More specific subject area**	Diagnostic immunochemistry
**Name of your method**	Optimization of immunohistochemistry by using biotinylated scFv
**Name and reference of original method**	This study builds upon the work of Rodriguez Nava et al. (2025), which involved the creation and functional validation of a recombinant anti-REST single-chain variable fragment (scFv). This scFv was directly compared with the parental IgM and a commercial antibody, showing similar specificity and detection performance. While that research demonstrated the feasibility of using the scFv for detecting REST, the current methodology focuses on optimizing the immunohistochemical workflow through the chemical biotinylation of the scFv. This adjustment allows for a streamlined biotin–streptavidin–peroxidase detection strategy, which eliminates the need for additional labeling steps, thus reducing both experimental time and costs while improving reproducibility. Moreover, the smaller molecular size of the biotinylated scFv facilitates better diffusion in paraffin-embedded tissues, offering a more efficient and scalable approach for protein detection in cervical tissues.
**Resource availability**	*n/a*

## Background

**Fig. 1:**
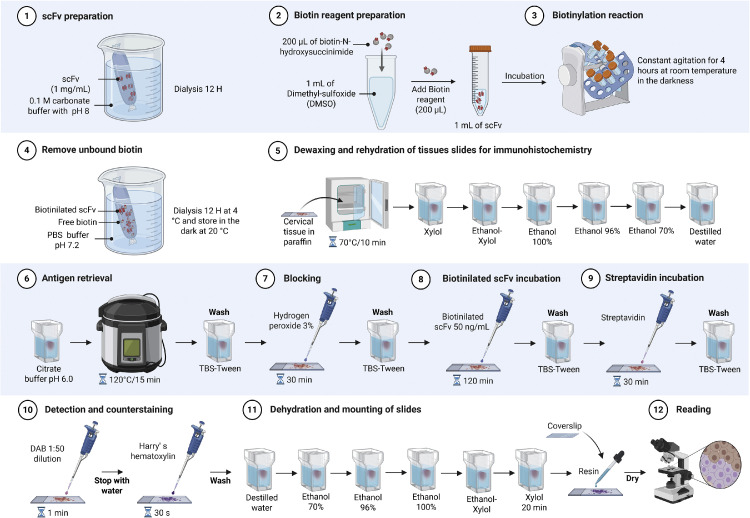
Schematic representation of the optimized immunohistochemistry method based on biotinylated scFv.

The proposed methodology aims to optimize and standardize the biotinylation and immunohistochemistry processes using scFv (Single-Chain Variable Fragment) (Fig. 1). This approach arises from the need to develop more specific, reproducible, and cost-effective methods for antigen detection in biological tissues, particularly in paraffin-embedded samples, where antigen preservation is particularly challenging. Traditional immunohistochemical techniques rely on full-length IgG antibodies, which, despite their widespread use, have significant limitations, including high production costs, batch-to-batch variability, and reduced tissue penetration [1]. In contrast, scFvs offer several advantages due to their small size, high binding affinity, and ease of production. The biotinylation of scFv could enhance their potential for sensitive and stable detection via the biotin–streptavidin system, thereby ensuring strong, reliable signal amplification [2].

The incentive behind this methodology lies in integrating all the necessary steps for obtaining a functional biotinylated scFv and applying it to histological antigen detection. Each phase—from scFv preparation, biotin conjugation, removal of the unbound reagent, and subsequent immunohistochemical application— was carefully designed to minimize antibody activity loss and reduce non-specific background staining. Additionally, the inclusion of antigen retrieval and blocking steps ensures improved specificity and clarity under microscopic visualization. A key advantage of this approach is that the biotinylated scFv eliminates the need to add biotin, shortens experimental time, reduces overall costs, and simplifies the workflow, making the process more efficient and accessible. The reduction in incubation stages also minimizes potential assay-to-assay variability, enhancing the reproducibility and reliability of the results [3].

Beyond its technical benefits, this methodology provides a versatile, adaptable platform for diverse tissue types and antigen targets. Also, represents a valuable alternative for basic research and diagnostic applications, where accurate biomarker detection is essential. The use of biotinylated scFvs advances sustainable, standardized immunodiagnostic techniques by reducing dependence on expensive, time-consuming commercial full-length antibodies. In summary, this methodology combines the sensitivity of the biotin–streptavidin system with the specificity and efficiency of scFv fragments, resulting in a robust, cost-effective, and adaptable approach for advanced immunohistochemical studies. By streamlining experimental procedures and reducing reagent use, it provides researchers with a practical, precise molecular tool for reliable, reproducible diagnostic and research applications [4].

## Method details

**Required reagents and equipment**

**Biotinylation reagents:**

- Beaker;
- Eppendorf tubes 1.5 mL;
- Amber tubes;
- Carbonate buffer pH 8 (Na_2_CO_3_ + NaHCO_3_ at 0.1 M, pH 8.0,);
- Dialysis membranes MWCO 12 to 14 kDa (Spectrum Cat# 08-700-166);
- Purified scFv;
- Sterile Phosphate buffered saline (PBS) pH 7.2 (prepared in-house);
- Dimethyl- sulfoxide (Sigma Aldrich Cat # D8418);
- Biotin-N-hydroxysuccinimide (Sigma Aldrich Cat# 0035,013,720);
- Tubes shaker;
- Fridge;
- Magnetic stirrer and stir bars.

**Tissues sample:**

- Electrocharged slides 27 × 76 mm (Star Frost Adhesive Cat# A100018);
- Cervical tissues in paraffin;
- Incubator.

**Histological reagents:**

- Pascal's pot (Dako, Carpinteria, CA, USA);
- Micropipette and tips;
- Glass coplins;
- Heat-resistant coplins with lid;
- Metal slide staining racks;
- Containers for wash buffer;
- Xylene (Hycel CAS:1330-20-7) and xylene-ethanol (1:1);
- Pure ethanol (Hycel CAS:64-17-5), ethanol 96% and ethanol 70% (Both diluted in distilled water);
- Distilled water (Hycel Cat#6544);
- Antigen retrieval buffer (Citrate buffer pH 6.0, BiosB Cat# BSB 0023);
- PBS-Tween (prepared in-house);
- Absorbent paper;
- Humid chamber;
- Hydrogen peroxide 3% (BiosB Cat# SBS0001S);
- Biotinylated scFv;
- PBS pH 7.2 (prepared in-house);
- Streptavidin conjugated to HRP (BiosB Cat# SBS0001S);
- Chromogen DAB and DAB diluent (BiosB Cat# SBS0001S);
- Harry´s Hematoxylin (Hycel Cat#738).

**Mounting materials:**

- Resin Entellan (Merck, Cat# 107,961);
- Coverslips;
- Microscope.

**Protective equipment:**

- Gloves;
- Lab coat;
- Face mask.

**Procedure**

**Note:** The immunohistochemical technique is carried out in a place with airflow and extractors, and with the correct use of appropriate protective equipment to avoid contact and inhalation of hazardous solvents.

1. **scFv preparation**: The purified scFv (1 mg/mL) is dialyzed for 12 h in 0.1 M carbonate buffer pH 8 (30 mM Na_2_CO_3_ and 70 mM NaCO_3_H).
  **Note:** For this step, place the dialysis membrane with the scFv in the beaker filled with the carbonate buffer, and shake with a magnetic stir bar, maintaining a slight, constant movement.
2. **Preparation of the biotin reagent**: Dissolve 2 mg of biotin ester-N-hydroxysucinimide in 1 mL of DMSO.
3. **Biotinylation reaction**: Add 200 µL of the biotin reagent to the previously dialyzed scFv and incubate for 4 h at room temperature under constant agitation in the dark to allow covalent conjugation. A ∼33-fold molar excess of biotin ester-N-hydroxysucinimide relative to scFv was used to favor efficient labeling while minimizing excessive lysine modification that could potentially interfere with antigen binding.
4. **Removal of Unbound Biotin:** Excess unbound biotin was removed by performing dialysis for 12 h at 4 °C using a 12–14 kDa molecular weight cut-off (MWCO) dialysis membrane. The buffer-to-sample ratio used was 50:1, meaning 50 mL of PBS for each 1 mL of sample. The dialysis buffer was replaced the following day, and dialysis was continued for an additional 20 min to enhance buffer exchange efficiency. Collect the dialyzed antibody in a new Eppendorf tube and store it in the dark at −20 °C until use. **Note:** PBS was prepared by dissolving 8.5 g (137 mM) of NaCl, 1.15 g (8.1 mM) of Na₂HPO₄, and 0.24 g (1.7 mM) of NaH₂PO₄·H₂O in 1 L of distilled water. The pH was adjusted to 7.2.
5. **Deparaffinization of cervical tissues:** Paraffin-embedded slides should be placed in an incubator at 70 °C until the paraffin becomes visibly liquid (approximately 15 min). Immediately after, transfer the slides to a Coplin bottle containing 100% xylene. **Note:** Avoid exposure to air during this step to prevent paraffin from solidifying.
6. **Rehydration of Cervical Tissues:** The tissues on the slides should be placed in metal slide staining racks and gradually rehydrated by passing through the following solutions: 100% xylene, xylene-ethanol (1:1), pure ethanol, 96% ethanol, 70% ethanol, and finally water. **Note:** Perform this step carefully to prevent detachment of the tissue.
7. **Antigen Retrieval:** Histological samples will undergo heat-induced antigen retrieval using 20X ImmunoDNA Retriever Citrate buffer (BiosB cat# BSB 0023), which must be diluted to 1X in distilled water before use. The antigen retrieval process should be conducted at pH 6.0 at 120 °C for 15 min. Allow the slides to cool to room temperature before removing them from the Coplin bottle. **Note:** Wash the tissue slides with PBS-Tween (0.05% Tween (Sigma-Aldrich Cat# P1379) in PBS) and carefully dry the area around the sample. Remove any residual buffer with absorbent paper to avoid diluting the next reagent. Perform five washes of three minutes each with TBS-Tween.
8. **Blocking:** To block endogenous peroxidase activity, incubate the tissue sections with 3% hydrogen peroxide (H₂O₂) for 30 min at room temperature in humidity chambers. Follow this with five washes of three minutes each using TBS-Tween.
9. **Incubation with Biotinylated scFv:** The biotinylated scFv should be added to reach a final concentration of 50 ng/mL and incubated for 120 min at room temperature, followed by another five washes of three minutes each with TBS-Tween.
10. **Incubation with Streptavidin–HRP Conjugate:** Slides will be incubated with horseradish peroxidase (HRP)-conjugated streptavidin for 30 min. Additional washes should be performed to remove excess reagent, with five washes of three minutes each using TBS-Tween.
11. **Detection and Counterstaining:** Antigen-antibody binding will be visualized using diaminobenzidine (DAB) as a chromogen, diluted 1:50 in DAB diluent. The chromogenic reaction should be developed for 30 s and then stopped with distilled water. Apply Harry’s hematoxylin for 30 s as a counterstain, followed by rinsing in distilled water to remove any excess dye.
12. **Dehydration and Mounting:** Remove residual water gradually by immersing the slides in the following solutions: 70%, 96%, and 100% ethanol, ethanol-xylene (1:1), and 100% xylene, with each step lasting 20 min. The slides should then be mounted using Entellan resin and coverslips and allowed to dry at room temperature.
13. **Microscopic Evaluation:** The stained sections will be examined under a Leica DM1000 LED optical microscope (Leica Biosystems, Wetzlar, Germany) using 20 × and 40 × objectives to assess signal localization and immunostaining intensity.

## Method validation

**Fig. 2:**
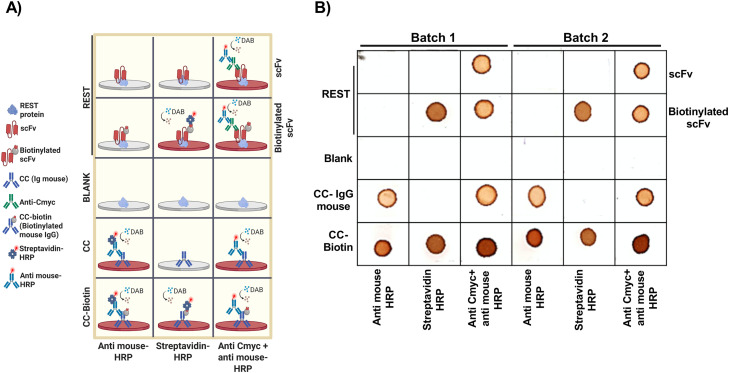
Dot blot validation of scFv biotinylation. (A) Schematic representation of the Dot blot assay used to verify scFv biotinylation and detection strategies. (B) Dot blot results obtained using two independent batches of biotinylated scFv (L1 and L2). Brown signals indicate positive detection after HRP-mediated DAB development. Biotinylated scFv incubated with immobilized REST and revealed with streptavidin–HRP produced positive signals, whereas non-biotinylated scFv and blank controls were negative. Additional positive and conjugate controls (CC: Total Ig´s of mouse and CC-Biotin; Biotinylated IgG of mouse) confirmed the detection specificity.

The biotinylation of the anti-REST single-chain variable fragment (scFv) was evaluated using Dot blot analysis, with immobilized REST protein serving as the antigen on a nitrocellulose membrane (Bio-Rad Cat # 1620,115). The experimental design included several detection strategies and controls to assess the presence of biotin on the scFv and to confirm the specificity of the detection system (Fig. 2A). Two independent batches of biotinylated scFv (L1 and L2) were analyzed in parallel to evaluate the reproducibility of the results (Fig. 2B). When REST protein was spotted on the membrane and incubated with the biotinylated scFv, followed by streptavidin conjugated to horseradish peroxidase (HRP, Sigma-Aldrich Cat# OR03L), a distinct brown signal was observed after the development with DAB (Sigma-Aldrich Cat# D8001) in both batches. In contrast, when non-biotinylated scFv was used under the same conditions, no signal was detected. This indicates that signal generation in this configuration relied on the presence of biotin in the scFv. Blank controls, where the primary antibody was omitted, did not produce any detectable signals, which helps rule out the possibility of a non-specific signal arising from the detection reagents. To further confirm that the scFv can bind to the antigen independently of biotinylation, membranes were incubated with the scFv and then treated with an anti-c-Myc antibody (produced in mice, Santa Cruz Biotechnology, Cat# sc-40), followed by an HRP-conjugated anti-mouse IgG (Jackson ImmunoResearch, Cat# 115-035-003) as a secondary antibody. Positive signals obtained using this method indicate that the scFv retains its antigen-binding activity and that the c-Myc tag introduced during cloning remains accessible after biotinylation. Conjugate controls were included to assess the specificity of the detection systems. Dots containing non-biotinylated mouse IgG (CC) and biotinylated mouse IgG (CC-Biotin, prepared in-house) displayed the expected signal patterns: both were detected by anti-mouse HRP, while only the CC-Biotin produced a signal when revealed with streptavidin–HRP. Additionally, Dots incubated with non-biotinylated scFv and revealed without the intermediate anti-c-Myc antibody showed no signal. This result is consistent with the absence of an Fc region in the scFv fragments, confirming that observed signals in the assay do not arise from direct recognition by the HRP-conjugated secondary antibodies. In summary, the Dot blot results support the suitability of the biotinylated scFv for streptavidin-based detection. This technical validation formed the foundation for subsequent immunohistochemistry experiments utilizing a streptavidin–HRP detection system with the biotinylated scFv.

## Application of the biotinylated scFv in immunohistochemistry

**Fig. 3:**
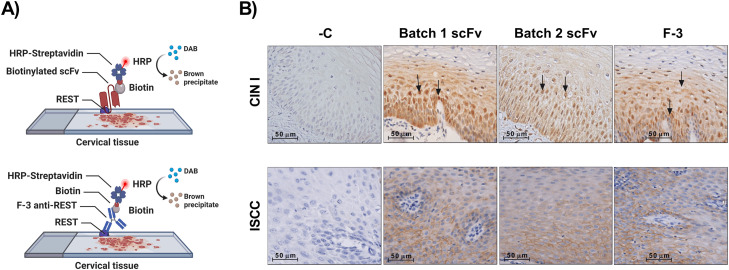
Immunohistochemical Detection of REST. (A) This panel illustrates the biotin–streptavidin–peroxidase immunohistochemistry principle using an optimized configuration with a biotinylated single-chain variable fragment (scFv) (upper section) alongside a commercial anti-REST monoclonal antibody (F-3, Santa Cruz Biotechnology, Cat# sc-374,611; lower section). (B) This panel shows representative immunohistochemical staining of cervical tissue sections from cases of cervical intraepithelial neoplasia grade I (CIN I) and invasive squamous cell carcinoma (ISCC). The staining utilized two independent batches of purified biotinylated scFv (Batch 1 and Batch 2) as well as the commercial F-3 antibody. Negative controls (-C) were processed simultaneously, with the primary antibody omitted. Black arrows highlight areas with strong REST immunoreactivity. The signal was visualized using HRP-mediated 3,3′-diaminobenzidine (DAB) development. Scale bar = 50 µm.

The biotinylated single-chain variable fragment (scFv), validated through Dot blot analysis, was subsequently used in an immunohistochemistry (IHC) protocol that employed a biotin-streptavidin-peroxidase detection system. Panel A provides a schematic overview of the detection principles. The conventional IHC method using a commercial anti-REST monoclonal antibody (F-3 from Santa Cruz Biotechnology, Cat#sc-374,611) is presented as a reference. In contrast, the optimized method with the biotinylated scFv is illustrated as a simplified detection scheme, where antigen recognition and signal amplification are directly linked through streptavidin-HRP (Fig. 3A). Fig. 3B displays representative immunohistochemical staining obtained from cervical tissue sections. Two independent batches of purified biotinylated scFv (Batch 1 and Batch 2) were evaluated alongside the commercial F-3 antibody under identical experimental conditions. In cervical intraepithelial neoplasia grade I (CIN I) samples, immunoreactivity was observed in both the nuclear and cytoplasmic compartments using both the biotinylated scFv and the commercial antibody. In samples of invasive squamous cell carcinoma (ISCC), staining was predominantly found in the cytoplasm. Negative controls, processed without the primary antibody, showed no detectable staining, confirming the specificity of the detection method. The staining patterns were comparable between the two independent batches of biotinylated scFv and the commercial antibody, indicating reproducible antigen recognition across different preparations. Furthermore, the biotinylated scFv allowed for direct detection with streptavidin-HRP, thus eliminating the necessity for an intermediate secondary antibody and streamlining the staining workflow. Together with the Dot blot validation experiments, these findings support the use of the biotinylated scFv as a detection reagent for streptavidin-based immunohistochemical analysis of REST expression in cervical tissues (Fig. 3B).

## Limitations

The biotinylation reaction depends on the chemical coupling to accessible lysine residues, which may result in inconsistent labeling among scFv molecules. Variations in biotinylation efficiency, such as over-biotinylation or under-biotinylation, can impact antigen binding and reduce detection efficiency. Therefore, it is essential to validate each batch, for instance, through Dot blot analysis.

## Ethics statements

Cervical samples were collected from women who had previously given informed consent, in accordance with the Helsinki Declaration of 2013. The ethics committee of the Dirección de Investigación at the Universidad Autónoma de Guerrero approved the use of these samples (General Agreement UAGro-IECan 04/18/2016).

## CRediT author statement

**Cynthia Rodriguez-Nava and Karen Cortés-Sarabia**: Conceptualization, Investigation, Methodology, Validity tests, Data curation, Writing- Original draft preparation. **Carlos Ortuño-Pineda, Berenice Illades-Aguiar, Amalia Vences-Velázquez and Luz del Carmen Alarcón-Romero**: Supervision, Validation, Writing- Reviewing and Editing.

## Declaration of competing interest

The authors declare that they have no known competing financial interests or personal relationships that could have appeared to influence the work reported in this paper.

## Data Availability

No data was used for the research described in the article.
